# Coffee Consumption and Heart Rate Variability: The Brazilian Longitudinal Study of Adult Health (ELSA-Brasil) Cohort Study

**DOI:** 10.3390/nu9070741

**Published:** 2017-07-13

**Authors:** Rackel Aguiar Mendes de Oliveira, Larissa Fortunato Araújo, Roberta Carvalho de Figueiredo, Alessandra C. Goulart, Maria Ines Schmidt, Sandhi Maria Barreto, Antonio Luiz Pinho Ribeiro

**Affiliations:** 1 Hospital das Clinicas and School of Medicine, Universidade Federal de Minas Gerais, Belo Horizonte 30130-100, Brazil; knutri2@gmail.com (R.A.M.d.O.); larissafortunatoaraujo@gmail.com (L.F.A.); robertafigueiredo@ufsj.edu.br (R.C.d.F.); sandhi.barreto@gmail.com (S.M.B.); 2 Universidade Federal de SãoJoão Del Rei, Divinópolis 35501-296, Brazil; 3 Center for Epidemiological and Clinical Research, Hospital Universitário, Universidade de São Paulo, São Paulo 05508-000, Brazil; agoulart@hu.usp.br; 4 Postgraduate Studies Program in Epidemiology, School of Medicine, Universidade Federal do Rio Grande do Sul, Porto Alegre 90040-060, Brazil; maria.schmidt@ufrgs.br

**Keywords:** coffee consumption, heart rate variability, coronary artery disease

## Abstract

Studies have shown that acute coffee ingestion can affect cardiovascular autonomic activity, although the chronic effects on heart rate variability (HRV) remain controversial. Method: A cross-sectional study with baseline data (2008–2010) from ELSA-Brasil cohort of 15,105 (aged 35–74), based in six Brazilian states. Coffee consumption in the previous 12 months was measured using the semi-quantitative food frequency questionnaire, and HRV was obtained through electrocardiographic tracings during 10 min at rest. Independent association between the frequency of coffee consumption “never or almost never”, “≤1 cup/day”, “2–3 cups/day”, “≥3 cups/day”, and HRV was estimated using generalized linear regression, adjusting for socio-demographic characteristics, health-related behavior, markers of abnormal metabolism, and the presence of coronary artery disease. Further, we applied Bonferroni correction in the full models. Results: The mean age was 52 years (standard deviation (SD) = 9.1), and 52% was female; 9.5% never/almost never consumed coffee. In univariate analysis, coffee consumers had reduced values of HRV indexes, but after full adjustments and correction for multiple comparisons, these associations disappeared. A trend of reduction in HRV vagal indexes was observed in those that consumed ≥3 cups of coffee/day. Conclusion: Most of the effects attributed to the chronic use of coffee on the HRV indexes is related to the higher prevalence of unhealthy habits in coffee users, such as smoking and alcohol use. Adjustment for confounding factors weaken this association, making it non-significant. The effect of higher daily doses of coffee on the autonomic system should be evaluated in further studies.

## 1. Introduction

Coffee is a very popular drink all over the world, not only for its organoleptic characteristics, but also for its stimulating effect [1], being the main source of caffeine (1,3,7-trimethylxanthine) [1,2]. The acute and chronic cardiovascular effects of coffee and caffeine on the cardiovascular system have been the subject of controversy in studies carried out over recent decades [2,3,4]. Among the acute effects, we can highlight an increase in blood pressure and an activation of the sympathetic nervous system, with an increase in circulating catecholamines [3]. On the other hand, chronic coffee consumption was primarily associated with increased cardiovascular risk [5]. However, recent studies indicate a predominantly beneficial effect—especially when coffee is consumed moderately and for a long period of time [6]. In previous studies performed in the Brazilian Longitudinal Study of Adult Health (ELSA-Brasil), coffee consumption in the preceding 12 months was associated with a lower incidence of newly-diagnosed diabetes in adults [7], and also with better cognitive performance in the elderly [8]—events related to cardiovascular health.

Heart rate variability (HRV) is a non-invasive method used to evaluate the autonomic modulation of cardiac activity [9]. HRV indexes are obtained by different methods based on variations in the duration of inter-beat (RR) intervals in the electrocardiogram, and can be used to assess the presence of autonomic imbalance and increased risk of death in healthy subjects and under different clinical conditions, such as acute myocardial infarction and heart failure [10,11]. There are few studies on the effects of coffee and caffeine on HRV, and results are inconsistent. Some studies showed an increase [12,13], while in others there was no change [14,15] or a reduction [16] in vagal indexes of HRV in response to the ingestion or infusion of coffee and caffeine, both in healthy individuals and in those who had suffered myocardial infarction [17], or had diabetes [18] or heart failure [19].

Therefore, this study intends to evaluate a large sample of adults and elderly people living in six Brazilian states to determine if coffee consumption in the previous 12 months is associated with HRV alterations, regardless of potential confounding factors, such as socio-demographic characteristics, health-related behavior, metabolic alteration markers, and the presence of coronary artery disease.

## 2. Materials and Methods

### 2.1. Study Design and Population

The Brazilian Longitudinal Study of Adult Health (ELSA-Brasil) is a prospective multi-center study of 15,105 civil servants aged 35–74, who are active and retired from higher education and research institutions located in six Brazilian states: Minas Gerais, Rio de Janeiro, São Paulo, Espírito Santo, Bahia, and Rio Grande do Sul. The main objectives of ELSA-Brasil are to investigate risk factors (biological, behavioral, environmental, occupational, psychological, and social) related to the incidence and progression of chronic non-communicable diseases—particularly cardiovascular diseases and diabetes. Baseline data were collected by trained and certified professionals between August 2008 and December 2010 using face-to-face interviews, as well as physical and laboratory exams. Details on the study design and cohort profile can be found in other publications [20,21].

This study included all ELSA-Brasil baseline participants (2008–2010), excluding individuals with no valid data for frequency of coffee consumption (*n* = 28) and those who did not complete the HRV test (*n* = 1835). As such, of the 15,105 participants, 13,270 composed the study population.

### 2.2. Heart Rate Variability

The HRV test was performed in the morning in an appropriate place with little ambient noise, adequate lighting, and adequate temperature. In preparation for the test, the skin was cleaned at the distal ends of the arms and legs (inner face) with a 70% alcohol solution and cotton for the proper placement of the electrodes. After these procedures were completed, participants remained at rest for 10 min for the acquisition of the electrocardiogram (ECG) tracing data, lying comfortably with their arms and legs stretched out, adopting the supine position, having been instructed not to speak or move for the duration of the test [22].

The electrocardiograph used for the HRV test was the Micromed device (Brasília, Brazil). We used the WinCardio software program (version 4.4a, by Micromed, Brasília, Brazil to automatically generate the interval series (intervals between QRS complexes, the ECG waveform corresponding to the contraction of the ventricles, in normal sinus depolarization) from a single ECG lead (generally D2) [22,23]. To measure HRV, indexes were obtained through linear methods, divided into two types: time domain analysis (time interval of each normal RR (sinus beats) calculated over a 10-min interval approximately and expressed in milliseconds (ms) or percentages); and analysis of frequency domain (density of spectral power expressed in ms^2^ by frequency range in Hertz) [9]. The time domain statistical indexes evaluated were: (1) standard deviation of normal RR expressed in ms (SDNN); (2) root mean square for the square of the differences between normal adjacent RR intervals, expressed in ms (RMSSD); (3) percentage of adjacent RR intervals with difference in duration greater than 50 ms (pNN50) [9] and (4) mean RR interval expressed in milliseconds. The frequency domains evaluated were: (1) high frequency component (HF), corresponding to spectral power density in the frequency range between 0.15 and 0.4 Hz; and (2) low frequency component (LF), corresponding to spectral power density in the frequency range 0.04 to 0.15 Hz [9].

### 2.3. Coffee Consumption

Coffee consumption was obtained through a semi-quantitative food frequency questionnaire (FFQ) containing 114 food items which evaluated food consumption in the previous 12 months, with moderate to good reproducibility [24]. Participants were asked to read a list of foods and answer how many times they consumed coffee per day, week, or month. The ELSA-Brasil FFQ was structured in three sections: food/preparation, portion consumption measurements, and frequency of consumption, with eight possible choices: “more than 3 times/day”, “2–3 times/day”, “once/day”, “5–6 times/week”, “2–4 times/week”, “once/week”, “1–3 times/month”, and “never/almost never” [24]. For the purposes of this analysis, coffee consumption was categorized into: “Never or almost never”, “≤1 cup/day”, “2–3 cups/day”, and “≥3 cups/day”, considering the quantity of coffee consumed each time was 50 mL.

### 2.4. Co-Variables

We selected the following variables, in addition to sex (men/women) and age (in years): (a) Educational attainment categorized as university graduate (>15 years), high school graduate (11–14 years), elementary school completed (8–10 years), and incomplete elementary school (0–7 years); (b) Occupational status categorized as active or retired; (c) Smoking, smokers being considered as those who responded that they have smoked at least 100 cigarettes over the course of their lives and continue to smoke. Those who reported having smoked during their lives, but who no longer smoked at the time of the interview were classified as former smokers; (d) Consumption of alcohol was defined by the type of drink usually consumed, as well as the frequency and patterns of consumption. Information gathered from the questionnaire was converted into grams of alcohol consumed per week. Those who did not consume or almost never consumed were grouped in the same category; moderate consumption was classified as up to 210 g of alcohol per week for men, and 140 g per week for women; those who consumed more than this were classified as excessive consumers; (e) Physical activity in leisure time information, gathered through the International Physical Activity Questionnaire (IPAQ) and classified as strong, moderate, and light. Moderate physical activity included those who performed intense physical activity ≥3 days for a period of 20 min/day; participants who exercised ≥3 days for 30 min/day at moderate intensity and/or walking; or >5 days, ≥600 MET-minute with walking intensity moderate or vigorous; or >3 days with ≥1500 MET-minute and vigorous intensity. In the strong physical activity group, we included those who exercised seven times per week, equivalent to ≥3000 MET-minute with walking intensity moderate or strong. Individuals who did not meet the criteria for moderate or strong activity were classified in the light physical activity group. A MET-minute is computed by multiplying the MET score (METs are multiples of the resting metabolic rate) by the minutes performed; (f) Body mass index (BMI) (kg/m^2^); (g) Presence of diabetes defined by at least one of the following criteria: prior diagnosis reported by participant or use of medication for the treatment of diabetes, fasting glycemia ≥126 mg/dL, or glucose tolerance test ≥200 mg/dL or glycated hemoglobin ≥6.5%; (h) Systolic blood pressure (SBP) was measured using a validated oscillometric device (Omron HEM 705CPINT). SBP measurements were taken three times in the seated position after 5 min at rest, with the mean of the second and third measurements analyzed; (i) The ratio of total cholesterol to high-density lipoprotein (HDL-cholesterol) determined by standard enzymatic methods; (j) Presence of coronary artery disease defined by prior medical diagnosis of acute myocardial infarction or myocardium revascularization or heart failure or presence of higher Q waves on the electrocardiogram.

### 2.5. Statistical Analysis

The difference between groups according to frequency of coffee consumption was evaluated by means of chi-square tests (categorical variables), ANOVA (continuous variables with symmetrical distribution), or Kruskal–Wallis (continuous variables with asymmetric distribution). The chi-square test for trend (Dunn Test) was used to verify the presence of a possible linear relationship between the frequency of coffee consumption and HRV indexes.

We investigated the association between the frequency of coffee consumption and each response variable by using generalized linear models (GLMs). GLMs are an extension of classic linear regression models. The estimation of parameters in GLMs is done by the maximum likelihood method, and the generalization of GLMs creates a range of options for the distribution of the response variable, dispensing with the requirements of normality, linearity, and homoscedasticity for data analysis [25]. For a better interpretation, the coefficients of the GLMs were exponentiated representing the arithmetic mean ratios (AMRs).

Initially, we investigated the association between coffee consumption and each HRV index (model 1), before adjusting for sex, age, educational attainment, and occupational status (model 2); sequentially, we introduced the variables of smoking, alcohol consumption, physical activity, body mass index, diabetes, systolic blood pressure, total ratio/HDL-cholesterol (model 3); finally, there was an adjustment for coronary artery disease (model 4). In the full model, we applied the Bonferroni correction [26,27] to the values shown in the final multivariate models to account for the multiplicity of statistical tests performed in the present analysis (model 5). The magnitudes of the associations were estimated by the AMR and their 95% confidence intervals. Analyses were carried out using the Stata 12.0 (Stata Corporation, College Station, TX, USA).

### 2.6. Ethical Aspects

ELSA-Brasil was approved by the Research Ethics Committees of participating education and research institutions, and by the National Research Ethics Committee (CONEP 976/20060) of the Ministry of Health. All participants signed the Free and Informed Consent Form before the interviews, examinations, and measurements were carried out.

## 3. Results

The mean age of the study population was 52 years (standard deviation (SD) = 8.9), and 52.3% were women. Among the total number of participants, 1261 (9.5%) never or almost never consumed coffee; 4266 (32.1%) consumed ≤1 cup per day; 4616 (34.8%) consumed 2 to 3 cups per day; and 3127 (23.6%) consumed ≥3 cups per day. The frequency of coffee consumption was statistically associated with all the characteristics of the study population included in Table 1, except the presence of diabetes and coronary artery disease (Table 1).

Table 2 describes the indexes of the HRV in frequency and time domains by coffee consumption frequency category. The RMSSD, pNN50, HF, LF, and total RR variance were associated with coffee consumption frequency. We noted the values of HRV indexes in the category of never/almost never consume coffee, and in the category of more frequent coffee consumption (≥3 cups/day). 

Table 3 shows the results of the regression analyses between coffee consumption frequency and the different HRV indexes. In the univariate analysis (model 1), in the time domain, in comparison to the participants who never/almost never consumed coffee, all consumption frequencies were associated with lower HRV (with the exception of mean RR interval), although without dose–response relation.

In the multivariate analysis, after adjusting for age, sex, educational attainment, and occupational status (model 2), and then for smoking, alcohol consumption, BMI, systolic blood pressure, diabetes, and total cholesterol/HDL ratio (model 3), the AMRs related to the categories of coffee consumption ≤1 cup per day but 2–3 cups/day lost statistical significance, especially between the spectral components (HF, LF). However, between the vagal indexes (RMSSD, pNN50, and HF), we noted RMAs of a moderate magnitude among those who consumed ≤1 cup/day (RMSSD and pNN50) and ≥3 cups/day (RMSSD, pNN50 and HF) in relation to the participants who never/almost never consumed coffee. For SDNN, the association with coffee consumption was statistically significant only for those who consumed ≤1 cup per day.

After adjustment for coronary heart disease (Model 4), the associations between categories of coffee consumption frequency and the individual indexes of HRV lost significance when compared to those who never/almost never consumed. Nevertheless, participants who consumed ≥3 cups per day maintained a RMSSD vagal index on average 4% lower when compared to those who never/almost never consumed coffee. Furthermore, we applied the Bonferroni correction in the full model, and coffee consumption did not remain associated with RMSSD (Model 5).

## 4. Discussion

In this large population study, after progressively adjusting for socio-demographic characteristics, health-related behaviors, metabolic alteration markers, and the presence of coronary artery disease, we noted that chronic coffee consumption loses its association with reduction of vagal control of the heart. Consumption of three cups or more of coffee in the previous 12 months was associated with levels 4% lower on average in the RMSSD index in the final model, although the association between categories of coffee consumption frequency and RMSSD loses significance when compared to those who never/almost never consume after correction for multiple comparisons using the Bonferroni method.

HRV alterations found in the univariate analysis, with reduction in all general HRV indexes, including the specific vagal indexes RMSSD, pNN50, and HF component, suggest a predominant reduction of vagal influence on heart rate. In the conditions under which the exams were carried out in the ELSA-Brasil study, at rest, the autonomic influence is predominantly parasympathetic, so all indexes reflect mostly a vagal influence [28]. Therefore, the findings of this univariate analysis could be interpreted as if the chronic consumption of coffee was associated with the inhibition of vagal autonomic modulation of heart rate—a phenomenon that was related to increased cardiovascular risk in community cohort studies [29].

Association of chronic coffee consumption with the reduction of some HRV indexes disappeared when we controlled for the effect of potential confounding factors in this association. When compared with those who never/almost never consumed coffee, chronic coffee consumers are older and more frequently moderate or excessive alcohol consumers, smokers, and sedentary. Advanced age is associated with a reduction in HRV, especially for vagal components, as well as alcohol consumption, smoking, and sedentary lifestyle [30,31]. The greater frequency of coffee use among smokers and consumers of alcohol seems to at least partially explain the now outdated concept that coffee is associated with increased risk of coronary artery disease [32,33].

It is interesting to note that even when the effect of the confounding factors is removed, there is still a trend of a reduction in HRV indexes RMSSD, PNN50, and HF among consumers of three or more cups of coffee, suggesting a loss of statistical power to recognize this association. These indexes assess the variability of successive heartbeats or in the high frequency range, and are considered to be almost exclusively vagal indexes. As such, it is possible that the alterations found in our study may show the inhibitory effect of coffee on more frequent consumers of the drink, suggesting that different doses could have distinct effects on study participants. Indeed, coffee is a complex drink, with various chemically-active substances other that caffeine, so the cardiovascular response may vary not only according to the dose, but also the concentration of the drink used (strong, moderate, or weak coffee, coffee with milk, etc.) and the form of preparation (espresso, strainer, others).

To our best knowledge, this study is the largest (in number of participants) to study the association between coffee consumption and HRV. Other strong points of this study are the standardized and strict collection of data and the possibility to adjust for several confounding factors. Among the limitations, we recognize the retrospective nature of the information-gathering—particularly for coffee consumption—which can lead to errors in measurement. The participant may not correctly remember her/his chronic consumption of food and drink and overestimate or underestimate the frequency of ingestion. Another concern is the potential effect of unmeasured confounders, such as other beverages and foods which contain caffeine and residual confounding that is inevitable in studies of this nature. Finally, the transverse character of the study prevents the creation of causal associations.

## 5. Conclusions

In summary, this study shows that the association between coffee consumption and HRV reduction disappears for HRV indexes after adjusting for potential confounding factors, but that the chronic use of coffee in high doses (three or more cups per day in the last year) may be related to a reduction in the indexes related to the vagal control of heart rate. This study sheds further light on a complex issue regarding the effects of coffee—which has a large consumption in human nutrition—on the activity of the autonomic nervous system. Prospective studies may help to ascertain the effect of coffee in different contexts and its possible clinical-epidemiological importance.

## Figures and Tables

**Table 1:** Characteristics of the study population (35–74 years) by coffee consumption frequency in the last 12 months in Brazilian Longitudinal Study of Adult Health (ELSA-Brasil) participants (2008–2010).

Variables	Coffee (Cups/Day)				
	Never or Almost Never (*n* = 1261)	≤1 Cup/Day (*n* = 4266)	2–3 Cups/Day (*n* = 4616)	≥3 Cups/Day (*n* = 3127)	*p*-Value
**Sex (women), %**	52.3	54.8	59.3	48.1	**<0.001**
**Age (years), mean (SD)**	50.4 (9.1)	52.2 (9.4)	52.7 (9.1)	51.3 (8.0)	**<0.001**
**Educational Attainment, %**					
Under graduate school or more	51.1	50.5	54.2	52.5	**<0.001**
Complete high school	37.2	35.7	34.0	34.0	
Complete elementary school	7.3	7.4	6.3	7.1	
Incomplete elementary school	4.0	6.4	5.5	6.4	
**Occupational Status, %**					
Active	9.9	30.9	33.8	25.4	**<0.001**
Retired	8.0	37.3	38.9	15.8	
**Alcohol Consumption, %**					
Never or almost never	43.1	30.5	30.1	26.7	**<0.001**
Moderate	51.2	61.6	63.6	63.6	
Heavy	5.6	7.9	6.3	9.7	
**Smoking Status, %**					
Never	70.3	60.5	60.4	42.5	**<0.001**
Former	23.4	30.0	29.7	32.2	
Current	6.3	9.5	9.8	25.3	
**Physical activity, %**					
Heavy	11.7	9.4	8.1	8.1	**<0.001**
Moderate	15.1	14.6	14.1	12.3	
Weak	73.1	76.0	77.7	79.6	
**BMI (Kg/m^2^), mean (SD)**	4.9	4.7	4.6	4.6	**0.007**
**Diabetes status, %**	19.7	20.3	19.0	18.9	0.385
**Systolic blood pressure (mmHg), mean (SD)**	121.1 (17.7)	122.3 (17.8)	121.4 (17.0)	120.1 (16.9)	**<0.001**
**Total/HDL-Cholesterol ratio (mg/deciliters), mean (SD)**	3.9 (1.0)	4.0 (1.0)	3.9 (1.1)	4.0 (1.0)	**<0.001**
**Coronary Heart Disease Status, %**	3.0	3.0	3.2	2.8	0.837

BMI = body mass index; *n* = number; % = percentage; and SD = standard deviation, HDL-cholesterol = high-density lipoprotein. Performed Chi-square for frequencies, ANOVA for normal distribution.

**Table 2:** Mean (standard deviation) and median (interquartile range) of heart rate variability (HRV) indexes for by coffee consumption frequency in the last 12 months in ELSA-Brasil participants (2008–2010).

HRV Indexes	Coffee (Cups/Day)					
	Never or Almost Never (*n* = 1261)	≤1 Cup/Day (*n* = 4266)	2–3 Cups/Day (*n* = 4616)	≥3 Cups/Day (*n* = 3127)	*p*-Value	*p*-Value Tendency ^c^
**Time domain**						
SDNN (ms) ^a^, mean (SD)	42.3 (17.7)	40.2 (17.3)	40.6 (16.7)	41.4 (17.0)	0.066	0.208
RMSSD (ms) ^b^, median (1° and 4° quartile)	27.0 (18.9–37.8)	24.7 (16.9–35.6)	24.8 (17.2–35.6)	25.7 (17.4–35.8)	<0.001	0.274
pNN50 (%) ^b^, median (1° and 4° quartile)	4.7 (0.9–15.1)	3.3 (0.5–12.3)	3.4 (0.5–12.7)	4.0 (0.6–12.9)	<0.001	0.354
Mean RR interval (ms) ^a^, mean (SD)	916.7 (125,2)	911.6 (126.7)	913.1 (127.3)	913.2 (122.3)	0.080	0.905
**Frequency domain**						
HF (ms^2^) ^b^, median (1° and 4° quartile)	262.3 (115.9–540.5)	211.0 (95.8–447.8)	218.4 (96.5–470.8)	223.4 (97.7–468.2)	<0.001	0.143
LF (ms^2^), median (1° and 4° quartile)	263.8 (122.7–498.3)	225.1 (106.5–454.4)	232.4 (105.1–481.6)	251.6 (117.0–530.8)	<0.001	0.164

^a^ ANOVA for normal distribution and ^b^ Kruskal–Wallis test for non-normal distribution, Dunn Test. Abbreviations: HRV = heart rate variability, SDNN = standard deviation of normal inter-beat (RR) intervals; RMSSD = root mean square for the square of the differences between normal adjacent RR intervals; pNN50 = percentage of adjacent RR intervals with difference in duration greater than 50 ms; HF = high frequency component; LF = low frequency component. ^c^ *p*-value tendency.

**Table 3:** Associations of coffee consumption frequency and heart rate variability indexes of participants (35 to 74 years of age) of ELSA-Brasil (2008–2010).

HRV Indexes	Coffee (Cups/Day)				
	Model 1 Coefficient (95%CI)	Model 2 Coefficient (95% CI)	Model 3 Coefficient (95% CI)	Model 4 Coefficient (95% CI)	Model 5 Coefficient (95% CI)
**Time domain**					
**SDNN (ms)**					
Never or almost never	Reference	Reference	Reference	Reference	Reference
≤1 cup/day	0.95 (0.93, 0.98) **	0.97 (0.94, 0.99) *	0.97 (0.95, 0.99) *	0.97 (0.95, 1.00)	0.97 (0.94, 1.01)
2–3 cups/day	0.96 (0.93, 0.98) **	0.98 (0.96, 1.01)	0.99 (0.96, 1.01)	0.99 (0.96, 1.02)	0.99 (0.96, 1.03)
≥3 cups/day	0.98 (0.95, 1.00)	0.98 (0.96, 1.01)	0.98 (0.96, 1.01)	0.99 (0.96, 1.01)	0.99 (0.95, 1.02)
**RMSSD (ms)**					
Never or almost never	Reference	Reference	Reference	Reference	Reference
≤1 cup/day	0.93 (0.90, 0.97) **	0.96 (0.92, 0.99) *	0.96 (0.92, 0.99) *	0.97 (0.93, 1.01)	0.97 (0.92, 1.02)
2–3 cups/day	0.94 (0.91, 0.98) **	0.97 (0.93, 1.00)	0.97 (0.94, 1.01)	0.98 (0.94, 1.02)	0.98 (0.93, 1.03)
≥3 cups/day	0.94 (0.91, 0.98) **	0.96 (0.92, 0.99) *	0.95 (0.91, 0.99) *	0.96 (0.92, 0.99) *	0.96 (0.91, 1.01)
**pNN50 (%)** ^a^					
Never or almost never	Reference	Reference	Reference	Reference	Reference
≤1 cup/day	0.87 (0.80, 0.95) **	0.91 (0.83, 0.99) *	0.92 (0.84, 1.00)	0.94 (0.85, 1.03)	0.95 (0.84, 1.06)
2–3 cups/day	0.88 (0.80, 0.96) **	0.94 (0.86, 1.03)	0.95 (0.87, 1.04)	0.97 (0.88, 1.07)	0.98 (0.87, 1.10)
≥3 cups/day	0.88 (0.80, 0.96) **	0.92 (0.84, 1.01)	0.90 (0.82, 0.99) *	0.92 (0.83, 1.02)	0.94 (0.83, 1.06)
**Mean RR interval (ms)**					
Never or almost never	Reference	Reference	Reference	Reference	Reference
≤1 cup/day	0.99 (0.98, 1.00)	0.99 (0.99, 1.00)	0.99 (0.99, 1.00)	0.99 (0.99, 1.01)	0.99 (0.98, 1.01)
2–3 cups/day	0.99 (0.98, 1.00)	0.99 (0.99, 1.01)	0.99 (0.99, 1.01)	1.00 (0.99, 1.01)	1.00 (0.99, 1.01)
≥3 cups/day	0.99 (0.98, 1.00)	0.99 (0.98, 1.00)	0.99 (0.98, 1.00)	0.99 (0.98, 1.00)	0.99 (0.98, 1.01)
**Frequency domain**					
**HF (ms^2^)**					
Never or almost never	Reference	Reference	Reference	Reference	Reference
≤1 cup/day	0.89 (0.82, 0.97) **	0.93 (0.85, 1.02)	0.94 (0.86, 1.03)	0.97 (0.88, 1.06)	0.97 (0.86, 1.09)
2–3 cups/day	0.88 (0.81, 0.96) **	0.94 (0.86, 1.02)	0.95 (0.87, 1.04)	0.97 (0.89, 1.076)	0.97 (0.87, 1.09)
≥3 cups/day	0.86 (0.79, 0.94) **	0.90 (0.82, 0.98) *	0.89 (0.81, 0.98) *	0.91 (0.832, 1.01)	0.92 (0.81, 1.04)
**LF (ms^2^)**					
Never or almost never	Reference	Reference	Reference	Reference	Reference
≤1 cup/day	0.92 (0.85,0.99) **	0.95 (0.88, 1.03)	0.95 (0.88, 1.03)	0.96 (0.88, 1.04)	0.96 (0.87,1.07)
2–3 cups/day	0.91 (0.85,0.98) **	0.98 (0.91, 1.06)	0.98 (0.91, 1.06)	1.01 (0.93, 1.10)	1.01 (0.91, 1.11)
≥3 cups/day	0.99 (0.91,1.07)	1.00 (0.93, 1.09)	0.99 (0.91, 1.07)	1.01 (0.92, 1.10)	1.01 (0.90, 1.02)

Performed generalized linear model (arithmetic mean ratio, AMR). Abbreviations: HRV = heart rate variability, SDNN = standard deviation of normal RR; RMSSD = root mean square for the square of the differences between normal adjacent inter-beat (RR) intervals; pNN50 = percentage of adjacent RR intervals with difference in duration greater than 50 ms; HF = high frequency component; LF = low frequency component. Model 1: Unadjusted model. Model 2: Adjusted for age, sex, educational attainment, and occupational status. Model 3: Model 2 + adjusted for smoking status, alcohol consumption, physical activity, body mass index, diabetes status, systolic blood pressure, and total/HDL-cholesterol ratio. Model 4: Model 3 + adjusted for coronary heart disease status. Model 5: Model 4 considering Bonferroni corrections. ** *p* ≤ 0.01 and * *p* > 0.01 and ≤ 0.05.
